# Preparation and Characterization of Poly(Acrylic Acid)-Based Self-Healing Hydrogel for 3D Shape Fabrication via Extrusion-Based 3D Printing

**DOI:** 10.3390/ma16052085

**Published:** 2023-03-03

**Authors:** Woohyeon Shin, Kyeongwoon Chung

**Affiliations:** 1 Department of Biofibers and Biomaterials Science, Kyungpook National University, Daegu 41566, Republic of Korea; 2 School of Materials Science and Engineering, Ulsan National Institute of Science and Technology (UNIST), Ulsan 44919, Republic of Korea

**Keywords:** poly(acrylic acid), extrusion-based 3D printing, hydrogel, self-healing polymer, rheological characteristics

## Abstract

The three-dimensional (3D) printing of hydrogel is an issue of interest in various applications to build optimized 3D structured devices beyond 2D-shaped conventional structures such as film or mesh. The materials design for the hydrogel, as well as the resulting rheological properties, largely affect its applicability in extrusion-based 3D printing. Here, we prepared a new poly(acrylic acid)-based self-healing hydrogel by controlling the hydrogel design factors based on a defined material design window in terms of rheological properties for application in extrusion-based 3D printing. The hydrogel is designed with a poly(acrylic acid) main chain with a 1.0 mol% covalent crosslinker and 2.0 mol% dynamic crosslinker, and is successfully prepared based on radical polymerization utilizing ammonium persulfate as a thermal initiator. With the prepared poly(acrylic acid)-based hydrogel, self-healing characteristics, rheological characteristics, and 3D printing applicability are deeply investigated. The hydrogel spontaneously heals mechanical damage within 30 min and exhibits appropriate rheological characteristics, including G′~1075 Pa and tan δ~0.12, for extrusion-based 3D printing. Upon application in 3D printing, various 3D structures of hydrogel were successfully fabricated without showing structural deformation during the 3D printing process. Furthermore, the 3D-printed hydrogel structures exhibited excellent dimensional accuracy of the printed shape compared to the designed 3D structure.

## 1. Introduction

Poly(acrylic acid) (PAA) is widely used in various fields, such as pharmacology [1], packaging [2], adhesives [3], and biomedical applications [4], due to its excellent biocompatibility [5], pH sensitivity [6], bonding strength [7], and barrier quality [8]. Owing to the carboxylic acid functional group of PAA, it is pH-responsive and shows broad potential for drug delivery systems [6]. Additionally, the superior bonding strength of crosslinked PAA exhibits applicability for medical adhesives. For example, Solhi et al. demonstrated a novel nanofiller through the graft-polymerization of acrylic acid onto nanoclay platelets to improve the mechanical properties of dental adhesives [7]. In addition, film-type PAA is also applied to food packaging because of its excellent hardness and barrier quality. Kim et al. prepared nanocomposite film with reinforced mechanical and gas barrier properties based on poly(vinyl alcohol) (PVA)/poly(acrylic acid) (PAA)-layered double hydroxide (LDH) [8]. Furthermore, PAA-based hydrogels have been widely researched based on their extensive potential for various applications such as biosensors [9], drug delivery systems [10], and e-skin devices [11]. Lim et al. demonstrated a biosensor for detecting hepatitis B core antigen (HBcAg) based on the swelling of PAA hydrogel caused by the competitive binding of HBcAg to its antibody (anti-HBc) [9]. Wang et al. designed pH-sensitive poly(acrylic acid)/chitosan hydrogels to control the release of the drugs amoxicillin and meloxicam. The designed PAA-based hydrogel showed different drug-release behaviors depending on the pH value or degree of crosslinking [10]. A three-dimensional artificial skin device is demonstrated with self-healing and touch-sensing properties based on PAA-based hydrogel. The artificial skin device demonstrated excellent touch-sensing capability by showing a large electronic signal contrast upon human finger contact [11].

The 3D printing of polymeric materials was largely investigated in various applications, including dental implants [12], artificial bone [13], and 4D printing based on the shape memory properties of materials [14,15,16]. Hydrogels, as an attractive candidate for 3D printing of polymeric materials, have garnered significant interest owing to their superior properties, such as biocompatibility, flexibility, and tunable functionalities in various fields, including tissue engineering [17,18], food and agriculture industries [19,20], drug delivery systems [21], e-skin [22,23], and wearable devices [24,25].

In particular, many researchers in the field of e-skin have actively studied various functional hydrogels and their three-dimensional (3D) printing applications. Abodurexiti et al. designed an artificial epidermis based on a composite hydrogel ink with multifunctional characteristics for sensing pressure, temperature, strain, and humidity. The hydrogel inks were composed of polyvinyl alcohol (PVA), silk fibroin, carbon nanotubes, and conducting polymer solutions and were utilized to fabricate simple mesh structures using extrusion-based 3D printing. Additionally, they demonstrated the fabrication of high-density flexible electronic circuits using 3D printing [26]. Heidarian et al. prepared a nano-polysaccharide self-healing hydrogel for use in flexible strain sensors. The mesh-shaped hydrogel structure showed superior strain-sensing functionality based on the relative resistance changes due to bending angles [27]. To monitor human motion, Tang et al. designed deoxyribonucleic acid/poly (*N*-hydroxyethyl acrylamide) (DNA/pHEAA) double-networked hydrogels for flexible strain sensors. The designed transparent hydrogels were shaped in a snowflake pattern using injection molding and exhibited rapid self-recovery properties, excellent fatigue resistance, and high toughness [28]. Zhang et al. prepared supramolecular nanofibrillar hydrogels and developed highly stretchable, elastic, and sensitive ionic devices for strain and pressure sensing. The prepared hydrogels were fabricated into starfish shapes using a mold, and a spider web shape based on the dispensing of the hydrogels [29]. Although there is a significant amount of literature demonstrating the potential of hydrogels and their applications, only a few examples of complex 3D hydrogels for e-skin applications have been studied. Furthermore, most cases demonstrate the fabrication of films [30,31] or simple structures, such as porous meshes [26,27].

To successfully realize the 3D structures via extrusion-based 3D printing, an understanding of the material design of hydrogels and their correlation with their various characteristics is imperative. In our previous work, we systematically investigated the correlation between the material design factors of hydrogels and their characteristics, including gelation feasibility, rheological characteristics, and 3D printing processability [32]. During the investigation, we tuned five different material design factors, including monomer concentration, crosslinker length and concentration, dynamic crosslinker concentration, and ion concentration. We figured out that material design hugely influences material characteristics, as well as 3D printing processability. For example, a crosslinked PAA hydrogel containing a high concentration of NaCl or a PAA-based hydrogel without crosslinkers can be 3D printed, but the 3D structure collapses due to its low storage modulus and high tan δ (Table 1). On the other hand, the PAA hydrogel with higher crosslinking density showed a limitation in successfully printing continuous lines under 3D printing conditions due to its high storage modulus (Table 1). Based on the previous investigation, we defined a material design window (G′ < 2500 Pa and tan δ < 0.2) for the successful extrusion-based 3D printing application of hydrogels [32].

However, in our previous work, we explored the variation in the material properties of the hydrogel by gradually modifying one design factor at a time. The combination of multiple-factor designs and their effect on a material’s property and 3D printing applicability is still vague, especially whether the provided materials design window is applicable to the different hydrogels for successful extrusion-based 3D printing. It would be an important issue of interest since there are cases in which we are enforced to increase the content of certain design factors, such as dynamic crosslinkers or ionic conductors, to provide enhanced functionality. In these cases, we need to tune multiple design factors at the same time to control the rheological properties of the hydrogel for 3D printing applications.

**Figure 1:**
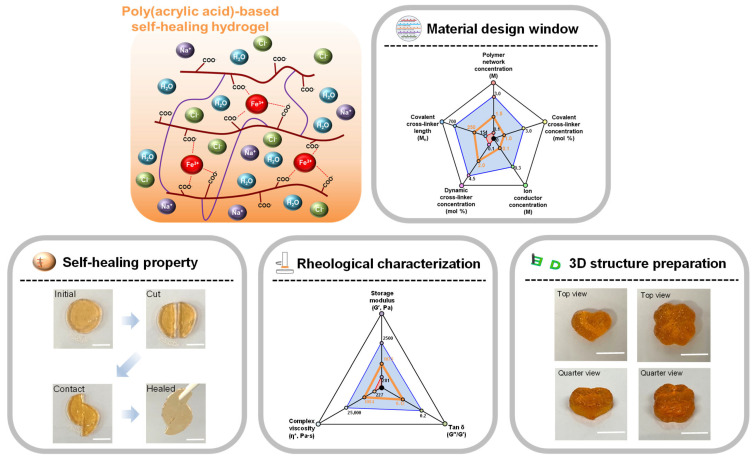
Schematic illustration of the prepared PAA-based self-healing hydrogel through material design, and investigation of its self-healing characteristics and rheological properties for 3D shape fabrication via extrusion-based 3D printing (scale bar: 10 mm).

Therefore, in this work, we prepared PAA-based self-healing hydrogel with a relatively high concentration of the dynamic crosslinker (FeCl_3_) which exhibits controlled rheological characteristics based on the modification of the crosslinker concentration. The self-healing characteristics, rheological characteristics, and 3D printing applicability is systematically investigated (Figure 1). The prepared hydrogel provided excellent self-healing characteristics by showing spontaneous self-healing in one piece within 30 min after a cut-and-contact process. The prepared hydrogel exhibited the rheological properties of G′~1075 Pa and tan δ~0.12, which shows an excellent fit for the previously defined materials design window (G′ < 2500 Pa and tan δ < 0.2) for extrusion-based 3D printing applications. With this, 3D-printed objects are successfully fabricated with excellent dimensional accuracy.

## 2. Materials and Methods

### 2.1. Materials

Iron chloride (FeCl_3_; Merck, Rahway, NJ, USA), sodium chloride (NaCl; Samchun Chemicals, Seoul, Republic of Korea), acrylic acid (AA, >99%; Tokyo Chemical Industry, Tokyo, Japan), poly(ethylene glycol) diacrylate (PEGDA, Mn∼250; Merck, Rahway, NJ, USA), and ammonium persulfate (APS; Samchun Chemicals, Seoul, Republic of Korea) were purchased and used without further purification.

### 2.2. Preparation of New Self-Healing Hydrogel

AA (1.5 M), PEGDA (15 mM), NaCl (0.1 M), and FeCl_3_ (30 mM) were added to deionized water and completely dissolved at room temperature, followed by argon bubbling for 30 min. APS (0.03 M), as an initiator, was then added to the pre-gel solution and stored in an oven at 50 °C for 2 h. The hydrogel was kept overnight at room temperature. After taking the gel out of the reactor, it was rinsed in 0.1 M NaCl solution to remove unreacted monomer, and then the surface of the gel was wiped off with filter paper.

### 2.3. Material Characterization

The new self-healing hydrogel was characterized with attenuated total reflection (ATR) mode using Fourier transform infrared spectroscopy (FT-IR, Nicolet 380, Thermofisher Scientific, Waltham, MA, USA). The FT-IR analysis was performed using completely dried hydrogel. The dried hydrogel was prepared by drying water under a vacuum at 80 °C.

### 2.4. Rheology Characterization

Rheological characterization of the new self-healing hydrogel was performed using a rheometer (MCR 92 and MCR 302; Anton Paar, Graz, Austria) at room temperature. Oscillatory shear was applied to the hydrogel loaded between two parallel plates for frequency sweep, amplitude sweep, and time-dependent recovery experiments. A frequency sweep was conducted in the angular frequency range of 0.1–10 rad/s under a constant shear strain of 5%. An amplitude sweep was performed in the strain range of 0.3–400% under a constant angular frequency of 1 rad/s while measuring the shear storage modulus (G′), shear loss modulus (G′′′), and complex viscosity (η*). The time-dependent recovery experiment was performed by maintaining a shear strain of 5% for 2 min, then applying a high shear strain of 400% for 1 min, and releasing it back to 5% for 10 min.

### 2.5. Three-Dimensional Printing of the New Self-Healing Hydrogel

Prior to 3D printing, the prepared hydrogel was placed in a 10 mL syringe and then centrifuged at 2000 rpm for 2–4 min to remove air bubbles. A metal nozzle with an inner diameter of 0.60 mm was set up on the syringe. The prepared syringe with the hydrogel sample was set up in extrusion-based 3D printers (a custom-made 3D printer; K Labs, Ulsan, Republic of Korea). To realize the fine 3D structure of the hydrogel, the printing conditions were controlled in the range of extrusion pressure of 150~300 kPa and 10~30% infill, and the printing speed was optimized at ~2 mm/s.

## 3. Results and Discussion

To obtain hydrogels with optimal physical and functional characteristics suitable for extrusion-based 3D printing, thorough material design is crucial in terms of various design factors including monomer concentration, crosslinker length and concentration, dynamic crosslinker concentration, and ion concentration. In our previous work, we found that the covalent crosslinker concentration is directly correlated with the physical properties of the hydrogel, and the concentration of the dynamic crosslinker is strongly associated with the self-healing properties as well as rheological characteristics [32]. Based on this relationship, we investigated how material design affects the material properties as well as their applicability in extrusion-based 3D printing, in case the covalent crosslinker and dynamic crosslinker concentration were adjusted simultaneously (Table 1). We prepared a new hydrogel based on a polyacrylic acid (PAA) network. PEGDA (1 mol% vs. AA monomer) was chosen as a covalent crosslinker, and multivalent iron ions (Fe^3+^) were utilized for secondary dynamic crosslinking based on ionic interactions between the multivalent cation and carboxylate anion (COO^−^) in the PAA chains (2 mol% vs. AA monomer). First, we prepared a pre-gel solution composed of AA (1.5 M), PEGDA (15 mM), FeCl_3_ (30 mM), and NaCl (0.1 M) in deionized water, and APS (0.03 M) was added as the initiator. The hydrogel was successfully prepared through free radical polymerization of PAA and PEGDA. The polymer network structure was characterized by Fourier transform infrared spectroscopy (FT-IR) (Figure S1).

**Figure 2:**
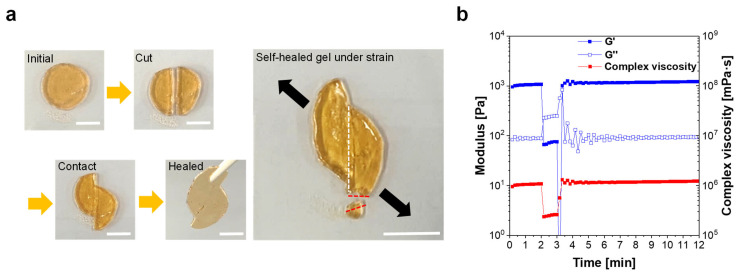
Characterization of the self-healing properties of the new PAA-based self-healing hydrogel. (**a**) Cut-and-contact self-healing test (scale bar: 10 mm). (**b**) Time-dependent recovery test of the hydrogel under varied shear strain conditions; 5% for initial 2 min, followed by 400% for 1 min, and 5% for 10 min (at 1 rad/s).

The self-healing characteristics of the hydrogels were examined using a cut-and-contact self-healing test [11]. In this test, the prepared hydrogel is cut into two pieces, which are then attached to each other at the cut interface and stored at room temperature for 30 min. The stored hydrogel recovers as one piece by reforming a secondary dynamic bonding network based on ionic interactions and metal–carboxylate coordination between the Fe^3+^ cations and carboxylate anions (COO^−^) (Figure 2a). Furthermore, when the self-healed hydrogel is elongated again, it is torn in a different direction (red dotted line, Figure 2a) compared to the formerly torn direction (white dotted line, Figure 2a). This observation strongly indicates that the prepared hydrogel efficiently recovers its mechanical properties by self-healing the polymer network.

Based on rheological characterization, the self-healing properties of the hydrogel were further analyzed via a time-dependent recovery test. The shear storage modulus (G′~1100 Pa) is much higher than the shear loss modulus (G′′~90 Pa) during the first 2 min (Figure 2b), exhibiting a viscoelastic solid-like behavior. However, G′ and G′′ are reversed under high shear strain, which represents viscoelastic liquid-like behavior resulting from the collapse of the polymeric network. After the shear strain is reduced back to 5%, the hydrogel rapidly recovers its G′, G″, and η* (in 0.5 min). It is worth noting that this excellent recovery characteristic is highly beneficial for extrusion-based 3D printing because it enables the hydrogel to maintain the 3D-printed shapes based on the rapidly regained viscoelastic solidity after being extruded from the 3D printer nozzle.

In our previous investigation [32], we demonstrated that the rheological characteristics of the hydrogel are highly important to successfully fabricate 3D structures via extrusion-based 3D printing. When the G′ of the hydrogel was higher than 2500 Pa, the rigidity of the gel increased, resulting in printed structures with disconnected hydrogel powders. Alternatively, when the tan δ (= G″/G′) of the hydrogels was higher than 0.2, the hydrogel became more flowable, and the printed structure started to rapidly collapse [32]. Based on this investigation, we presented a material design window in terms of rheological properties (G′ < 2500 Pa and tan δ < 0.2) for the successful extrusion-based 3D printing and structure fabrication of hydrogels.

**Figure 3:**
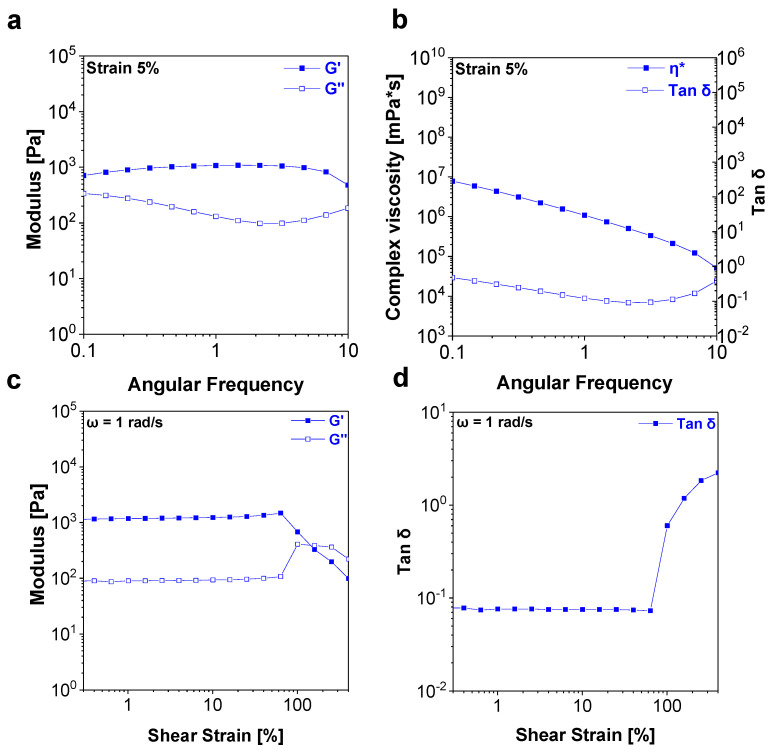
Rheological characterization of the new PAA-based self-healing hydrogel. (**a**,**b**) Frequency sweep and (**c**,**d**) amplitude sweep.

The prepared hydrogels in this study show viscoelastic solid-like behavior (G′ > G″) under a low angular frequency representing the stationary state and exhibit G′~1075 Pa and tan δ~0.12, which fit the suggested material design window. In addition, the complex viscosity (η*) of the hydrogel decreases as the applied angular frequency increases, showing shear-thinning behavior, which is favorable for extrusion-based 3D printing (Figure 3a,b) [33].

In the amplitude sweep (Figure 3c,d), the hydrogel exhibits viscoelastic solid-like behavior, G′ > G′′, under low shear strain, representing the stationary state, which is similar to the frequency sweep result. The hydrogel shows G′ > G′′ under applied strain (γ) less than 64%, which is indicative of the linear viscoelastic region. The G′ sharply decreased, and G″ approached G′ when γ > 64%, indicating the yielding of the hydrogel.

**Figure 4:**
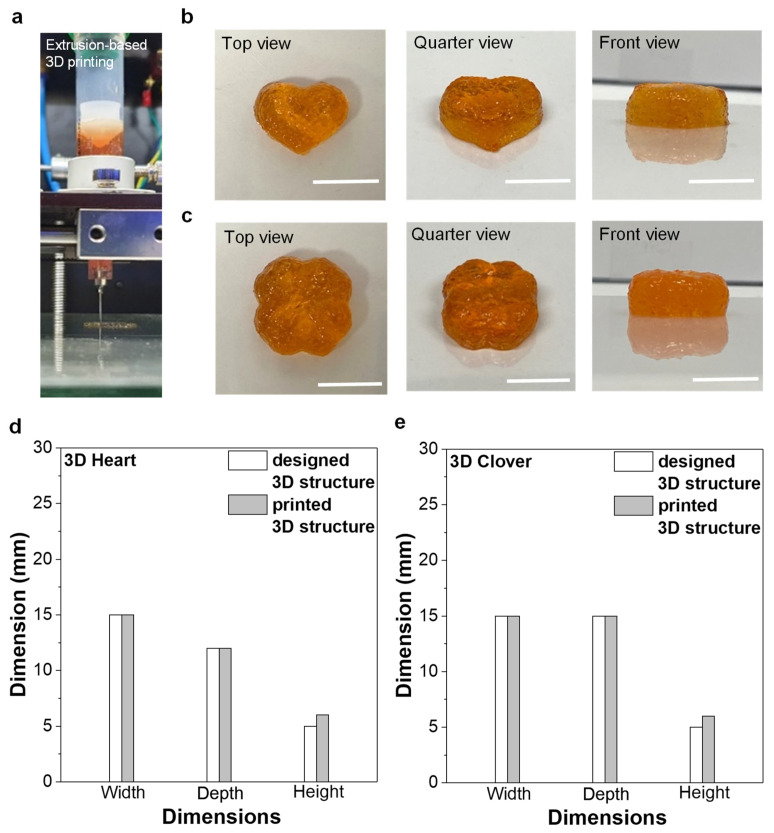
Three-dimensional printing of PAA-based self-healing hydrogel. (**a**) Photograph of extrusion-based 3D printer, (**b**,**c**) 3D-printed hydrogel structures in a heart shape (**b**) and a clover shape (**c**) (scale bar: 10 mm), (**d**,**e**) dimensional accuracy between the designed 3D structure and 3D-printed structure.

The prepared PAA-based self-healing hydrogel was subjected to an extrusion-based 3D printing process for the fabrication of complicated 3D structures. A custom-made extrusion-based 3D printer (Figure 4a) was used to extrude the materials using air pressure. Our 3D printing system is similar to direct ink writing, which is a simple method to realize various 3D structures based on the direct extrusion and deposition of several materials on a substrate. The printing system is generally equipped with a syringe with a fine nozzle for loading the sample; it is a dispenser that can move the syringe in the x-, y-, and z-directions. Various parameters, including applied pressure, nozzle size, moving speed, and the printing environment such as temperature and direct writing medium, directly affect the quality of 3D-printed objects [34].

Printing was conducted at room temperature without any support materials or additional hardening processes, such as post-UV curing or coagulation in a solution bath (Figure 4a). The hydrogel was successfully fabricated in various 3D structures, including ‘heart’ or ‘clover’ shapes without showing structural deformation during the 3D printing process (Figure 4b,c). The 3D-printed hydrogel structures exhibited excellent dimensional accuracy, showing almost identical dimensions for the width (W) and depth (D) and slightly increased dimensions in the height (H) of the printed shape compared to the designed 3D structure (Figure 4d,e). The slight deviation in size from the designed 3D structure is possibly due to the expansion of viscoelastic hydrogel upon extrusion from the narrow channel of the needle.

## 4. Conclusions

In this study, we successfully demonstrated a new PAA-based self-healing hydrogel by controlling the materials design factors such as the covalent and dynamic crosslinker concentrations simultaneously. The prepared new self-healing hydrogel exhibited fast recovery (~30 min) upon mechanical damage and is confirmed to show excellent applicability in extrusion-based 3D printing. From the rheological characterization, the prepared hydrogel showed rheological characteristics of G′~1075 Pa and tan δ~0.12, which accurately fit the previously defined material design window (G′ < 2500 Pa and tan δ < 0.2). The prepared hydrogel was directly applied to extrusion-based 3D printing without further treatment or post-processing after printing. The 3D objects exhibited excellent dimension accuracy compared to the designed 3D model. From the result, we can confirm that the defined material design window (G′ < 2500 Pa and tan δ < 0.2) in our previous work can serve as a guideline for fabricating extrusion-based 3D-printed functional hydrogels for various applications. We believe that this study will provide a better understanding of the preparation of hydrogels that are suitable for extrusion-based 3D printing through rheological characterization, and that this insight would be directly applicable to various functional hydrogels and their applications.

## Figures and Tables

**Table 1:** Materials design and their rheological properties for the prepared PAA-based self-healing hydrogel in this work and previously reported hydrogels [32] (reproduced with permission from Ref. [32], Copyright (2021), Wiley-VCH Verlag GmbH & Co. KGaA, Weinheim, Germany).

Sample	Acrylic Acid (M)	Covalent Crosslinker Length	Covalent Crosslinker Concentration (mol%/AA)	Dynamic Crosslinker Concentration (mol%/AA)	Ionic Conductor Concentration (M)	Storage Modulus (G’, Pa)	Tan δ	Reference
a	1.5	PEGDA (Mn~250)	1.0	2.0	0.1	1075	0.12	This work
b	1.5	PEGDA (Mn~250)	0.5	1.5	0.1	1055	0.081	[32]
c	1.5	PEGDA (Mn~250)	3.0	1.5	0.1	2806	0.041	[32]
d	1.5	PEGDA (Mn~250)	0.5	2.0	0.1	535	0.164	[32]
e	1.5	PEGDA (Mn~250)	1.0	1.5	0.1	1479	0.066	[32]
f	1.5	PEGDA (Mn~250)	0.5	1.5	0.3	124	0.393	[32]
g	1.5	PEGDA (Mn~250)	0	1.5	0.1	281	0.275	[32]

## Data Availability

Data presented in this study are available on request from the corresponding author.

## Supplementary Materials

The following supporting information can be downloaded at: https://www.mdpi.com/article/10.3390/ma16052085/s1, Figure S1: Chemical structure and characterization of a polymer network in the new self-healing hydrogel. (a), Chemical structure. (b), Fourier transform infrared (FT-IR) spectra [35].
