# Supplementary material for: Preparation and Characterization of Poly(Acrylic Acid)-Based Self-Healing Hydrogel for 3D Shape Fabrication via Extrusion-Based 3D Printing
Source: Materials (Basel). 2023 Mar 3;16(5):2085. doi: 10.3390/ma16052085 (PMC10004586; doi:10.3390/ma16052085)
Supplement: Supplementary file 1 [file materials-16-02085-s001.zip › materials-2169925-supplementary.pdf]

materials

Supporting Information

Preparation and characterization of poly(acrylic acid)-based self-healing hydrogel for 3D shape fabrication via extrusion-based 3D printing

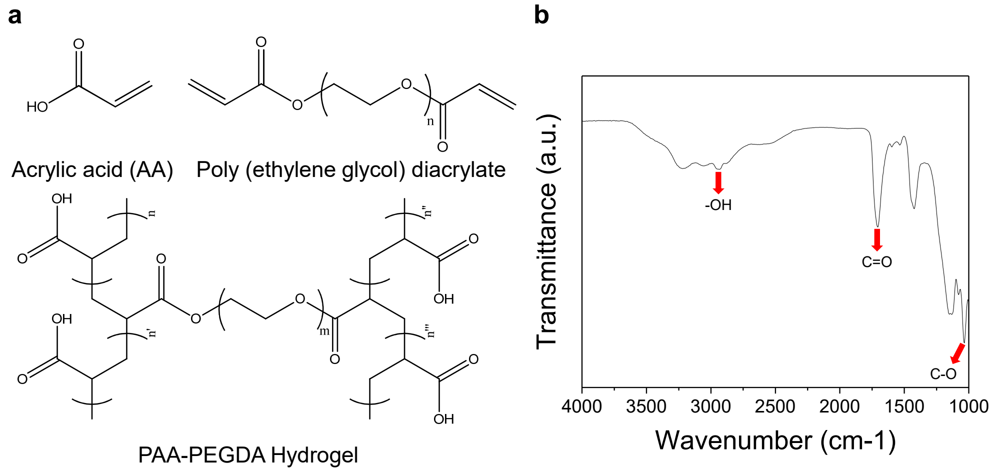

Figure S1. Chemical structure and characterization of polymer network in the new self-healing hydrogel. a, Chemical structure. b, Fourier transform infrared (FT-IR) spectra[1].

References

1. khoobi, M.; Moghimi, M.; Motlagh, G.H.; Sorouri, F.; Haririan, E. Cross-Linked Poly(Acrylic Acid) Hydrogel Loaded with Zinc Oxide Nanoparticles and Egg White Proteins for Antimicrobial Application. *J. Inorg. Organomet. Polym. Mater.* **2020**, *30*, 5234–5243.
